# The Garden and the Farm

**Published:** 1881-12

**Authors:** 


### THE GARDEN AND THE FARM.

### ARTIFICIAL DRYING OF CROPS.

The deterioration of the last British wheat crop, by reason of the heavy rains that fell just as the farmers were about to harvest it, brings into prominent notice an important invention for securing all agricultural products from the effects of dampness. This invention is the matured result of elaborate experiments by Mr. W. A. Gibbs, of Gilwell Park, Essex, England, and is known as the "Gilwell drier." Its essential feature is that hot air and the products of the combustion of coke or anthracite, as coming from a portable furnace, are driven by means of a fan right over and through the hay, wheat or other products to be cured. Under this process "the machine-dried hay," the *Journal of Science* says, "retains its full natural odor and savor and is eagerly eaten by the most fastidious cows and horses, and in practical working no disaster from fire or any other cause has ever occurred." Such an invention is invaluable, if, as this high scientific authority asserts, over thirty tons of wet hay can be dried by it in twenty-four hours at a cost less than four dollars, and that it is efficacious in saving grain, seed and all other products. The immense losses of British agriculturists from the saturation of their harvests by the proverbially torrential harvest rains have terribly crippled them for several years, so that some process for drying grain is a prime necessity. "The increasingly inferior condition in which English wheat is reaching the market is clearly shown," says the *Economist*, "by a comparison of the commercial statistics published during September" (the price falling from about fifty-five shillings a quarter on the 3d of the month to about forty-eight at its close), and "August was a month when the means of artificial drying would have been worth millions to British farmers." The value of Mr. Gibbs' machine might in some seasons be incalculable to our farmers, especially in the Eastern, Southern and Middle States, where wet weather in harvest is not unknown, and we see no reason why it might not be extensively employed in many other American farming districts where the summer rainfall is occasionally excessive and disastrous to the crops.

---

Courtesy is a powerful aid to him who receives. Treat even a base man with respect and he will make at least one desperate effort to be respectable.

